# Supplementary material for: Spermatozoal Mitochondrial Dynamics Markers and Other Functionality-Related Signaling Molecules Exert Circadian-like Response to Repeated Stress of Whole Organism
Source: Cells. 2022 Mar 15;11(6):993. doi: 10.3390/cells11060993 (PMC8946903; doi:10.3390/cells11060993)
Supplement: Supplementary file 1 [file cells-11-00993-s001.zip › cells-1608465-supplementary.pdf]

cells MDPI

# SUPPLEMENTARY

**Manuscript Title**

Spermatozoal mitochondrial dynamics markers and other functionality-related signaling molecules exert circadian-like response to repeated stress of whole organism

**Authors**

Isidora M Starovlah, Sava M Radovic Pletikosic, Tamara M Tomanic, Marija LJ Medar, Tatjana S Kostic, Silvana A Andric*

**Affiliation**

Laboratory for Reproductive Endocrinology and Signaling, Laboratory for Chronobiology and Aging, CeRES, DBE, Faculty of Sciences, University of Novi Sad, 21000 Novi Sad, Serbia

***Correspondence**

Silvana A Andric, PhD, professor
Head of Laboratory for Reproductive Endocrinology and Signaling
Member of Laboratory for Chronobiology and Aging
Head of accredited Center of Excellence for Reproductive Endocrinology
Faculty of Sciences, University of Novi Sad
Dositeja Obradovica Square 2, 21000 Novi Sad, Serbia
Tel. +381-21-485-2673 (office); +381-63-748-4403 (mobile); Fax: +381-21-450-620
E-mail: silvana.andric@dbe.uns.ac.rs
Web: http://wwwold.dbe.pmf.uns.ac.rs/en/nauka-eng/lares

Supplementary information file contains supplementary results and supplementary material and methods sections. Supplementary results section has presentation of the results from the main manuscript file calculated using the controls from ZT3 time point as calibrator. Material and methods section contains key resource table and tables of primers and antibodies used in the research.

## SUPPLEMENTARY RESULTS

**Relative expression of transcripts of mitochondrial biogenesis and functionality markers as well as markers of signaling pathways regulating mitochondrial dynamic and spermatozoa functionality calculated using the control group of the ZT3 time point as a calibrator.**

Calculation of relative gene expression, using the control group of the ZT3 time point as a calibrator, showed the changes of control and 10x3hIMO groups of each time point compared to the control group of the ZT3 time point. Transcriptional changes of mitochondrial dynamics and functionality markers in spermatozoa of repeatedly stressed adult rats are the most prominent at the ZT3 and ZT23 time points, compared to the ZT3-Controls. All of the followed mitochondrial biogenesis markers (except *Ppargc1b* and *Ppara*) and markers of mitochondrial fusion and architecture are significantly increased in spermatozoa from ZT3 and ZT23 10x3hIMO group compared to ZT3-Control group. In addition, *Drp1*, *Pink1*, *Prkn* and *Cox4i2* also increased, while *Ucp3* decreased, indicating significant transcriptional changes of mitochondrial autophagy and function at the ZT3 and ZT23 time point (Supplementary Figure S1, S2, S3, S4, S5). Also, transcription of the signaling molecules of cAMP and MAPK pathways (regulating mitochondrial dynamics and functionality as well as spermatozoa number and functionality) is the most significantly changed in the spermatozoa of repeatedly stressed adult rats at ZT3 and ZT23 time points, compared to the controls of the ZT3 time point (Supplementary Figure S6, S7).

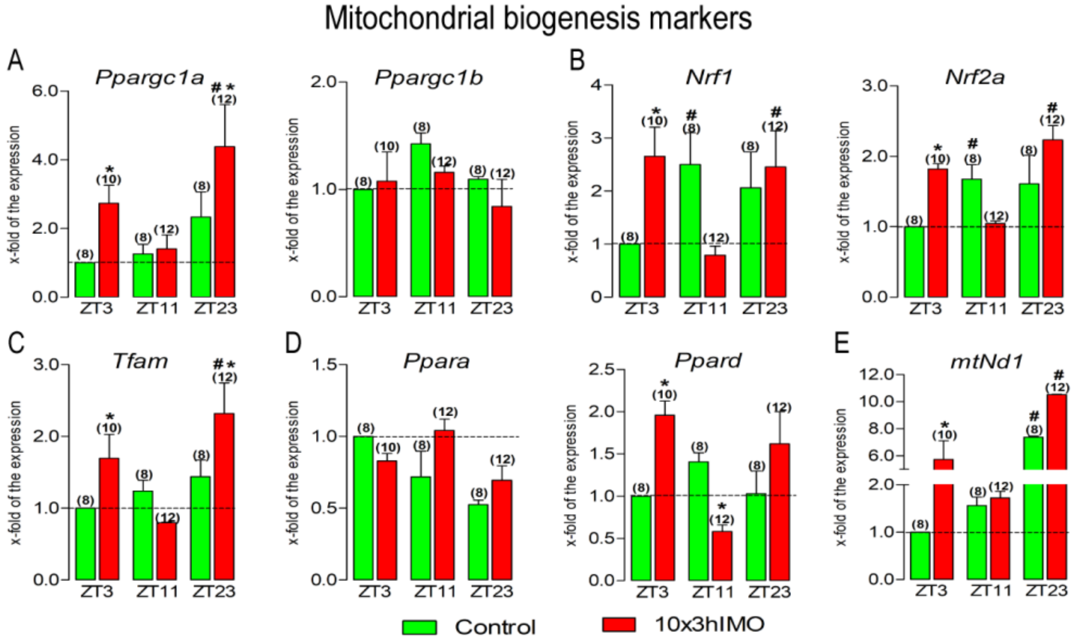


**Supplementary Figure S1.** Different transcriptional profiles of mitochondrial biogenesis markers in spermatozoa of repeatedly stressed adult rats at different time points normalized to the control group of the ZT3 time point.

Spermatozoa isolated from undisturbed and repeatedly stressed rats were used for RNA and protein isolation and further analysis of the transcriptional profile of mitochondrial biogenesis markers. Data bars are mean ± SEM values of two independent *in vivo* experiments. Statistical significance was set at level p <0.05: * vs. control group of the same time point, # vs. control group of the ZT3 time point.

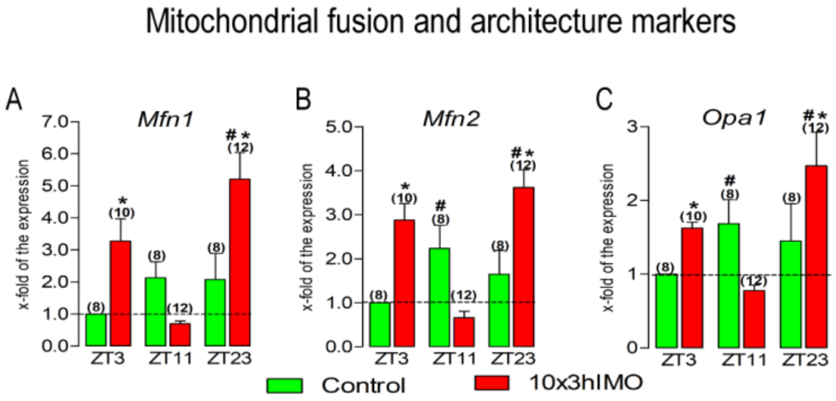


**Supplementary Figure S2.** Different transcriptional profiles of mitochondrial fusion and architecture markers in spermatozoa of repeatedly stressed adult rats at different time points normalized to the control group of the ZT3 time point. Spermatozoa isolated from undisturbed and repeatedly stressed rats were used for RNA isolation and further analysis of the transcriptional profile of markers of mitochondrial fusion and architecture. Data bars are mean ± SEM values of two independent *in vivo* experiments. Statistical significance was set at level $p < 0.05$: * vs. control group of the same time point, # vs. control group of the ZT3 time point.

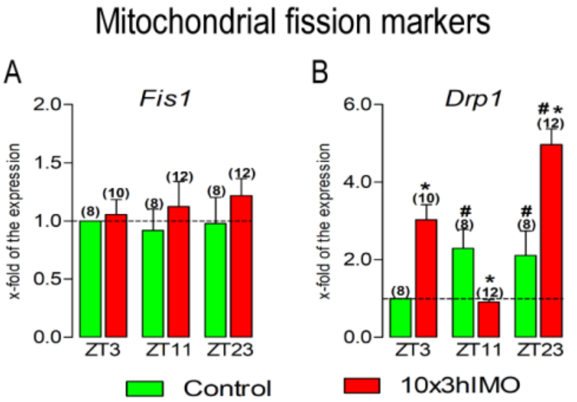


**Supplementary Figure S3.** Different transcriptional profiles of mitochondrial fission markers in spermatozoa of repeatedly stressed adult rats at different time points normalized to the control group of the ZT3 time point. Spermatozoa isolated from undisturbed and repeatedly stressed rats were used for RNA isolation and further analysis of the transcriptional profile of markers of mitochondrial fission. Data bars are mean ± SEM values of two independent *in vivo* experiments. Statistical significance was set at level $p < 0.05$: * vs. control group of the same time point, # vs. control group of the ZT3 time point.

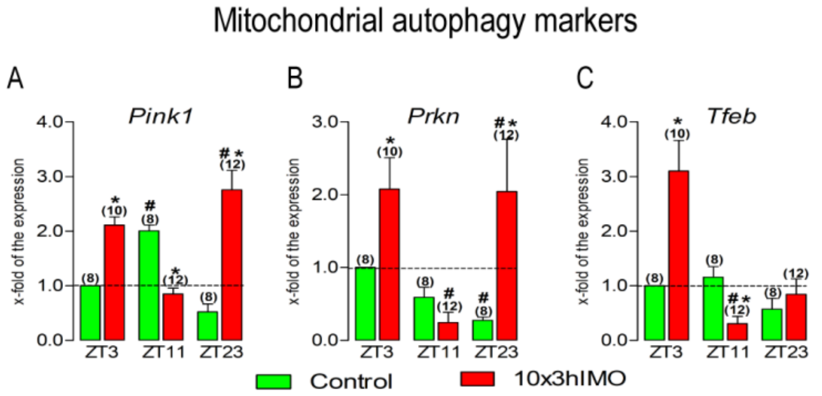


**Supplementary Figure S4.** Different transcriptional profiles of mitochondrial autophagy markers in spermatozoa of repeatedly stressed adult rats at different time points normalized to the control group of the ZT3 time point. Spermatozoa isolated from undisturbed and repeatedly stressed rats were used for RNA isolation and further analysis of the transcriptional profile of markers of mitochondrial autophagy. Data bars are mean ± SEM values of two independent *in vivo* experiments. Statistical significance was set at level p <0.05: * vs. control group of the same time point, # vs. control group of the ZT3 time point.

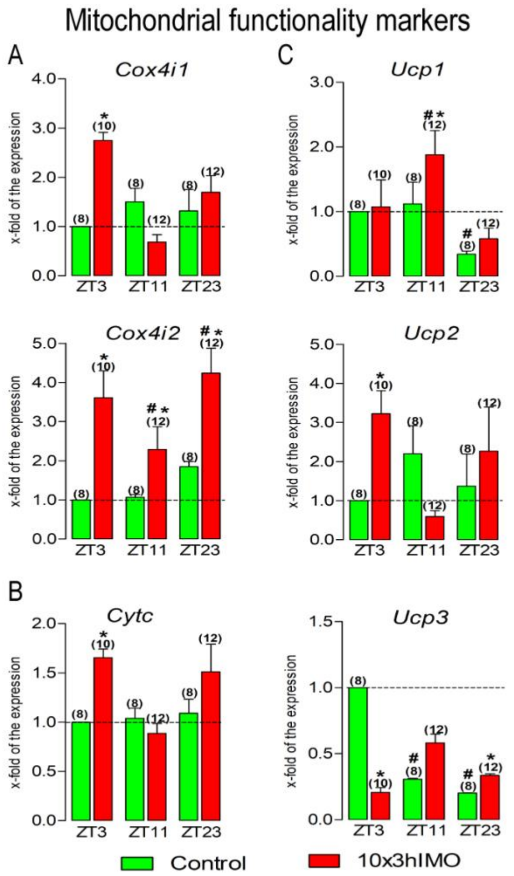


**Supplementary Figure S5.** Different transcriptional profiles of mitochondrial functionality markers in spermatozoa of repeatedly stressed adult rats at different time points normalized to the control group of the ZT3 time point. Spermatozoa isolated from undisturbed and repeatedly stressed rats were used for RNA isolation and further analysis of the transcriptional profile of markers of mitochondrial functionality. Data bars are mean ± SEM values of two independent *in vivo* experiments. Statistical significance was set at level p <0.05: * vs. control group of the same time point, # vs. control group of the ZT3 time point.

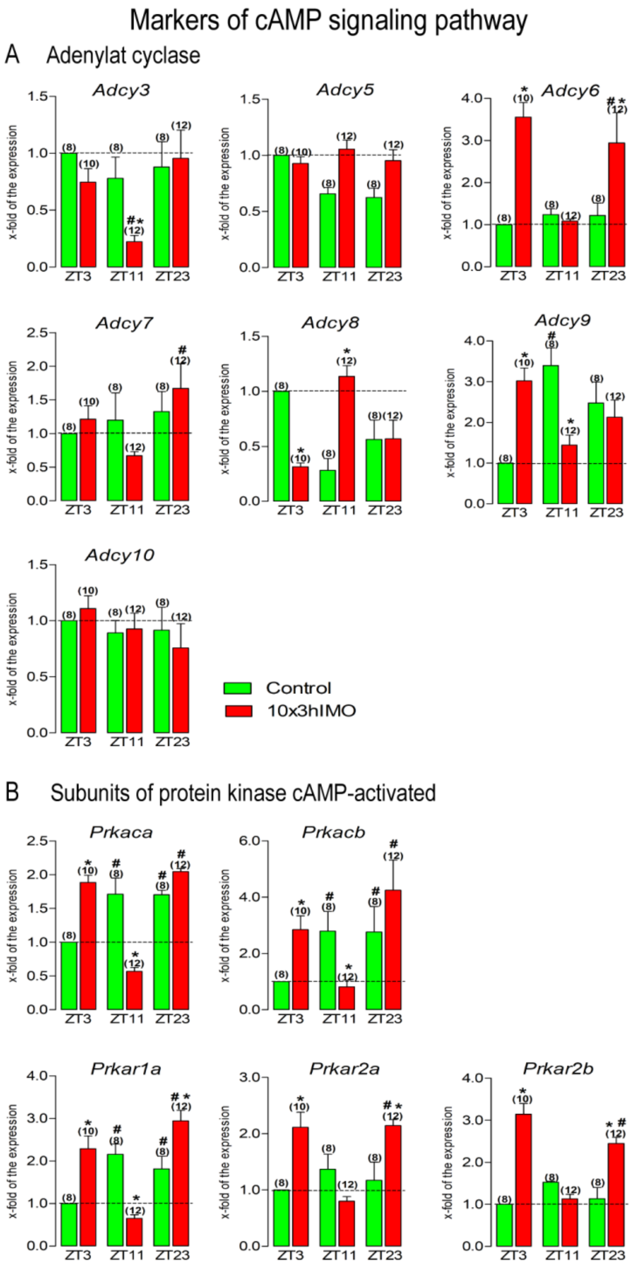


**Supplementary Figure S6.** Different transcriptional profiles of markers of cAMP signaling regulating mitochondrial dynamics and functionality as well as spermatozoa number and functionality in spermatozoa of repeatedly stressed adult rats at different time points normalized to the control group of the ZT3 time point. Spermatozoa isolated from undisturbed and repeatedly stressed rats were used for RNA isolation and further analysis of the transcriptional profile of markers of cAMP signaling pathway. Data bars are mean ± SEM values of two independent *in vivo* experiments. Statistical significance was set at level $p < 0.05$: * vs. control group of the same time point, # vs. control group of the ZT3 time point.

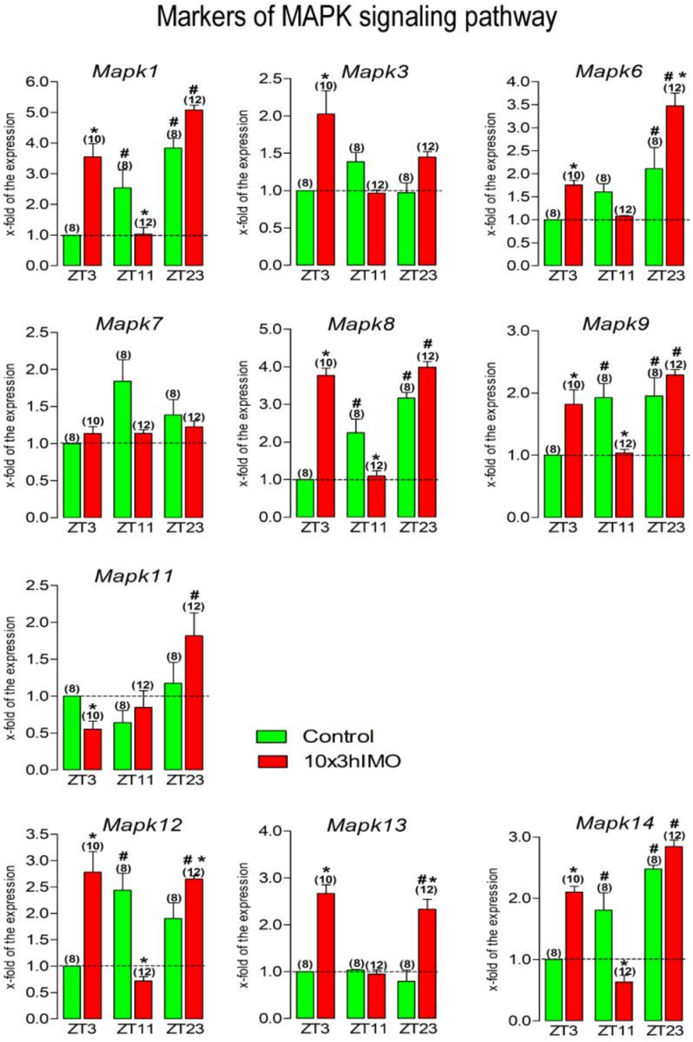


**Supplementary Figure S7.** Different transcriptional profiles of markers of MAPK signaling regulating mitochondrial dynamics and functionality as well as spermatozoa number and functionality in spermatozoa of repeatedly stressed adult rats at different time points normalized to the control group of the ZT3 time point. Spermatozoa isolated from undisturbed and repeatedly stressed rats were used for RNA isolation and further analysis of the transcriptional profile of markers of MAPK signaling pathway. Data bars are mean ± SEM values of two independent *in vivo* experiments. Statistical significance was set at level p <0.05: * vs. control group of the same time point, # vs. control group of the ZT3 time point.

## SUPPLEMENTARY MATERIAL AND METHODS

**Supplementary Table S1.** Key resources table.

| Resource or reagent | Source | Identifier |
|---|---|---|
| **Experimental model and biological samples** | | |
| *Wistar* rat | LaRES and ChronAge Laboratories (DBE, Faculty of Sciences, University of Novi Sad, Serbia) | http://wwwold.dbe.pmf.uns.ac.rs/en/nauka-eng/lares |
| Primary culture of spermatozoa | Three months-old male rats | NA |
| Serum | Three months-old male rats | NA |
| **Commercial Reagents/Assays** | | |
| Anti-testosterone serum №250 for Radioimmunoassay (T+DHT) | NA | NA |
| Corticosterone EIA Kit | Cayman Chemical, USA | https://www.caymanchem.com |
| GenElute™ Mammalian Total RNA Miniprep Kit | Sigma Aldrich, Germany | https://www.sigmaaldrich.com |
| DNase I (RNase-free) treatment | New England Biolabs, USA | https://international.neb.com |
| High Capacity Kit for cDNA | Applied Biosystems/Thermo Fisher Scientific, USA | https://www.thermofisher.com |
| Power SYBR® Green PCR Master Mix | Applied Biosystems/Thermo Fisher Scientific, USA | https://www.thermofisher.com |
| **Primers** | | |
| Supplementary Tables S2 to S8 | This paper | www.ncbi.nlm.nih.gov/sites/entrez |
| **Antibodies** | | |
| Rabbit polyclonal anti-PGC-1 (H-300) antibody | Santa Cruz Biotechnology Inc. | Cat # sc-13067 |
| Rabbit polyclonal anti-NRF2 (C-20) | Santa Cruz Biotechnology Inc. | Cat # sc-722 |

| | | |
|---|---|---|
| antibody | | |
| Mouse monoclonal anti-PKA[C] | BD Transduction Laboratories | Cat # 610980 |
| Rabbit polyclonal anti-p38MAPK antibody | Cell Signaling Technology | Cat # 9212 |
| **Software** | | |
| GraphPad Prism 5 Software | GraphPad Prism | https://www.graphpad.com/scientific-software/prism |

**Suppplementary Table S2.** Primers sequences used for the real-time PCR analysis of molecular markers of mitochondrial biogenesis.

| Gene | Accession code | Primers | Primer length | Product length | AV Ct | | |
|---|---|---|---|---|---|---|---|
| | | | | | ZT3 | ZT11 | ZT23 |
| ***Ppargc1a*** | NM_031347 | F: 5'-AGCCGTAGGCCCAGGTATGACA-3'<br>R: 5'-TGCTTGGCCCTTTCAGACTCCC-3' | 22 bp<br>22 bp | 107 bp | 30.32 | 31.06 | 31.47 |
| ***Ppargc1b*** | NM_176075 | F: 5'-ACCTTCCGGTGTTCGGAGCATG-3'<br>R: 5'-GTGGAAGGAGGGCTCATTGCGT-3' | 22 bp<br>22 bp | 81 bp | 28.64 | 28.63 | 29.07 |
| ***Tfam*** | NM_031326 | F: 5'-TATAGTCGTCGGCCCGAGGGAT-3'<br>R: 5'-AAGGCTGACAGGCGAGGGTATG-3' | 22 bp<br>22 bp | 125 bp | 28.32 | 28.62 | 28.46 |
| ***Nrf1*** | NM_001100708 | F: 5'-GACCATCCAGACGACGCAAGCA-3'<br>R: 5'-ATGGGCGGCAGCTTCACTGTT-3' | 22 bp<br>21 bp | 136 bp | 28.31 | 27.86 | 30.28 |
| ***Nrf2a*** | NM_001108841 | F: 5'-AGCGGAACTGAACCGCTTGGT-3'<br>R: 5'-GTGACTGGCTGAGCAATCCCGT-3' | 21 bp<br>22 bp | 84 bp | 28.02 | 27.55 | 28.27 |
| ***Ppara*** | NM_013196 | F: 5'-GTCCTGGAACTGAAGCGACGCT-3'<br>R: 5'-TTACGCCCAAATGCACCACGC-3' | 22 bp<br>21 bp | 110 bp | 28.36 | 28.86 | 27.77 |
| ***Ppard*** | NM_013141 | F: 5'-ACGGTAAAGGCGGTCCATCTGC-3'<br>R: 5'-TCCTCCTGTGGCTGTTCCATGAC-3' | 22 bp<br>23 bp | 109 bp | 26.63 | 26.29 | 27.62 |
| ***mtNd1*** | | F: 5' GCGTGGGAGGAGCATCAGGG 3'<br>R: 5' GCGAATGGTCCTGCGGCGT A 3' | 20 bp<br>20 bp | 271 bp | 18.44 | 18.73 | 20.00 |
| ***Gapdh*** | NM_017008 | F: 5'-TGCCAAGTATGATGACATCAAGAAG-3'<br>R: 5'-AGCCCAGGATGCCCTTTAGT-3' | 25 bp<br>20 bp | 110 bp | 21.42 | 21.25 | 21.23 |

Primers were designed by using software Primer Express 3.0 (Applied Biosystems) and full genes sequences from the NCBI Entrez Nucleotide database (www.ncbi.nlm.nih.gov/sites/entrez). F - forward; R - reverse.

**Supplementary Table S3.** Primers sequences used for the real-time PCR analysis of molecular markers of mitochondrial fusion and architecture.

| Gene | Accession code | Primers | Primer length | Product length | AV Ct | | |
|---|---|---|---|---|---|---|---|
| | | | | | ZT3 | ZT11 | ZT23 |
| ***Mfn1*** | NM_138976.1 | F: 5'-CCTTGTACATCGATTCCTGGGTTC-3'<br>R: 5'-CCTGGGCTGCATTATCTGGTG-3' | 24 bp<br>21 bp | 143 bp | 29.33 | 29.49 | 31.27 |
| ***Mfn2*** | NM_130894.4 | F: 5'-TCAAGCGCCAGTTTGTGGAG-3'<br>R: 5'-CACAGATGAGCAAATGTCCCAGA-3' | 20 bp<br>23 bp | 118 bp | 27.50 | 27.42 | 29.87 |
| ***Opa1*** | NM_133585.3 | F: 5'-AAAAGCCCTTCCCAGTTCAGA-3'<br>R: 5'-TACCCGCAGTGAAGAAATCCTT-3' | 21 bp<br>22 bp | 101 bp | 26.37 | 26.55 | 27.07 |
| ***Gapdh*** | NM_017008 | F: 5'-TGCCAAGTATGATGACATCAAGAAG-3'<br>R: 5'-AGCCCAGGATGCCCTTTAGT-3' | 25 bp<br>20 bp | 110 bp | 21.42 | 21.25 | 21.23 |

Primers were designed by using software Primer Express 3.0 (Applied Biosystems) and full genes sequences from NCBI Entrez Nucleotide database (*www.ncbi.nlm.nih.gov/sites/entrez*). F - forward; R - reverse.

**Supplementary Table S4.** Primers sequences used for the real-time PCR analysis of molecular markers of mitochondrial fission.

| Gene | Accession code | Primers | Primer length | Product length | AV Ct | | |
|---|---|---|---|---|---|---|---|
| | | | | | ZT3 | ZT11 | ZT23 |
| ***Fis1*** | NM_001105919.1 | F: 5'-ACGCCTGCCGTTACTTCTTC-3'<br>R: 5'-GCAACCCTGCAATCCTTCAC-3' | 20 bp<br>20 bp | 108 bp | 29.29 | 29.57 | 28.47 |
| ***Drp1*** | NM_053655.3 | F: 5'-AGGTTGCCCGTGACAAATGA-3'<br>R: 5'-CACAGGCATCAGCAAAGTCG-3' | 20 bp<br>20 bp | 94 bp | 30.05 | 29.92 | 32.32 |
| ***Gapdh*** | NM_017008 | 5'-TGCCAAGTATGATGACATCAAGAAG-3'<br>R: 5'-AGCCCAGGATGCCCTTTAGT-3' | 25 bp<br>20 bp | 110 bp | 23.16 | 21.31 | 20.83 |

Primers were designed by using software Primer Express 3.0 (Applied Biosystems) and full genes sequences from NCBI Entrez Nucleotide database (*www.ncbi.nlm.nih.gov/sites/entrez*). F - forward; R - reverse.

**Supplementary Table S5.** Primers sequences used for the real-time PCR analysis of molecular markers of mitochondrial autophagy.

| Gene | Accession code | Primers | Primer length | Product length | AV Ct | | |
|---|---|---|---|---|---|---|---|
| | | | | | ZT3 | ZT11 | ZT23 |
| ***Pink1*** | NM_001106694.1 | F: 5'-CAAGCAAGTGTCTGACCCAC-3'<br>R: 5'-GCTTCATACACAGCGGCATT-3' | 20 bp<br>20 bp | 111 bp | 26.18 | 25.25 | 27.67 |
| ***Prkn*** | NM_020093.1 | F: 5'-CTTCCAGCTCAAGGAAGTGG-3'<br>R: 5'-CAGAGGCATTTGTTTCGTGA-3' | 20 bp<br>20 bp | 182 bp | 32.70 | 33.43 | 34.79 |
| ***Tfeb*** | NM_001025707.1 | F: 5'-CGACAACATTATGCGCCTGG-3'<br>R: 5'-CTGTACACGTTCAGGTGGCT-3' | 20 bp<br>20 bp | 102 bp | 29.53 | 29.70 | 30.38 |
| ***Gapdh*** | NM_017008 | F: 5'-TGCCAAGTATGATGACATCAAGAAG-3'<br>R: 5'-AGCCCAGGATGCCCTTTAGT-3' | 25 bp<br>20 bp | 110 bp | 21.45 | 21.61 | 21.51 |

Primers were designed by using software Primer Express 3.0 (Applied Biosystems) and full genes sequences from NCBI Entrez Nucleotide database (*www.ncbi.nlm.nih.gov/sites/entrez*). F - forward; R - reverse.

**Supplementary Table S6.** Primers sequences used for the real-time PCR analysis of molecular markers of mitochondrial functionality.

| Gene | Accession code | Primers | Primer length | Product length | AV Ct | | |
|---|---|---|---|---|---|---|---|
| | | | | | ZT3 | ZT3 | ZT3 |
| ***Cox4i1*** | NM_017202 | F: 5'-CGCTGAGATGAACAAGGGCACC-3'<br>R: 5'-TCCCAGATCAGCACAAGCGCA-3' | 22 bp<br>21 bp | 93 bp | 22.55 | 22.48 | 24.20 |
| ***Cox4i2*** | NM_053472 | F: 5'-CACAGCCCAGGAAGTGCTGCTA-3'<br>R: 5'-TGTGCAGTAAGGCTCATCCGGC-3' | 22 bp<br>22 bp | 105 bp | 31.34 | 32.60 | 32.20 |
| ***Cytc*** | NM_012839 | F:5'-GCAAGCATAAGACTGGACCAAA-3'<br>R:5'-TTGTTGGCATCTGTGTAAGAGAATC-3' | 22 bp<br>25 bp | 88 bp | 23.62 | 23.85 | 23.87 |
| ***Ucp1*** | NM_012682 | F: 5'-TCAGCTCTTGTCGCCGGGTTT-3'<br>R: 5'-TGCACAGCTGGGTACACTTGGG-3' | 21 bp<br>22 bp | 114 bp | 29.77 | 30.06 | 29.85 |
| ***Ucp2*** | NM_019354 | F: 5'-ACGACCTCCCTTGCCACTTCAC-3'<br>R: 5'-GGTACTGGCCCAAGGCAGAGTT-3' | 22 bp<br>22 bp | 117 bp | 23.06 | 22.54 | 24.87 |
| ***Ucp3*** | NM_013167 | F: 5'-TGCTCAACCCACGGATGTGGT-3'<br>R: 5'-CCTGGCGATGGTTCTGTAGGCA-3' | 21 bp<br>22 bp | 112 bp | 28.97 | 29.80 | 29.71 |
| ***Gapdh*** | NM_017008 | F: 5'-TGCCAAGTATGATGACATCAAGAAG-3'<br>R: 5'-AGCCCAGGATGCCCTTTAGT-3' | 25 bp<br>20 bp | 110 bp | 21.42 | 21.25 | 21.23 |

Primers were designed by using software Primer Express 3.0 (Applied Biosystems) and full genes sequences from the NCBI Entrez Nucleotide database (*www.ncbi.nlm.nih.gov/sites/entrez*). F - forward; R - reverse.

**Supplementary Table S7.** Primers sequences used for the real-time PCR analysis of cAMP signaling elements.

| Gene | Accession code | Primers | Primer length | Product length | AV Ct | | |
|---|---|---|---|---|---|---|---|
| | | | | | ZT3 | ZT3 | ZT3 |
| ***Adcy3*** | NM_130779 | F: 5'-GCATCGAAACCTACCTCATCA-3'<br>R: 5'-TGGGCTCCTTGGTCTCA ATAA-3' | 21 bp<br>21 bp | 141 bp | 31.87 | 32.16 | 32.45 |
| ***Adcy5*** | NM_022600 | F: 5'-AACCAGGTGAACGCATGTCA-3'<br>R: 5'-CTCTGGGAAGTTGCAGTTGGA-3' | 20 bp<br>21 bp | 105 bp | 30.09 | 30.37 | 30.56 |
| ***Adcy6*** | NM_012821 | F: 5'-CTGCCTCAGCCTGCTTATGTG-3'<br>R: 5'-GGAGTCCTGGCGGAAGCT-3' | 21 bp<br>18 bp | 99 bp | 28.40 | 27.96 | 27.68 |
| ***Adcy7*** | NM_053396 | F: 5'-TTCCGTGCGTGTAACCCGCT-3'<br>R: 5'-GCCTTCTGCCTCCGTCCGTT-3' | 20 bp<br>20 bp | 123 bp | 27.54 | 26.72 | 26.92 |
| ***Adcy8*** | NM_017142 | F: 5'-ATTGCCTCAGTGGTGACTA-3'<br>R: 5'-CAAACTCTCCTCGGGCT-3' | 19 bp<br>17 bp | 113 bp | 29.21 | 30.71 | 29.01 |
| ***Adcy9*** | NM_001106<br>980 | F: 5'-TCACCAAGCTGTACGCCCGG-3'<br>R: 5'-GGGCTGTCAACACGTCCCGA-3' | 20 bp<br>20 bp | 124 bp | 30.41 | 29.40 | 29.17 |
| ***Adcy10*** | NM_021684 | F: 5'-CCAGGCATCGTGACCTGCGA-3'<br>R: 5'-ACTGGTCCGGGATCCGCAAC-3' | 20 bp<br>20 bp | 113 bp | 30.83 | 30.81 | 30.59 |
| ***Prkaca*** | NM_001100<br>922.1 | F: 5'-TCAGTGAGCCCCACGCCCGTT-3'<br>R: 5'-TCTCGGGCTTCAGGTCCCGG-3' | 21 bp<br>20 bp | 99 bp | 27.51 | 26.71 | 27.29 |
| ***Prkacb*** | NM_001077<br>645 | F: 5'-GGGTCATGGGGAACACGGCG-3'<br>R: 5'-CCAGCATTACTCGGGGGAGGGT-3' | 20 bp<br>22 bp | 124 bp | 28.76 | 27.46 | 27.73 |
| ***Prkar1a*** | NM_013181 | F: 5'-TGTGCTGCAGCGTCGGTCAG-3'<br>R: 5'-AGTGGCAGCCCGAGGACGAT-3' | 20 bp<br>20 bp | 112 bp | 25.94 | 24.74 | 24.63 |
| ***Prkar2a*** | NM_019264 | F: 5'-GCCCGACCTCGTCGACTTCG-3'<br>R: 5'-TCCTGCGCGTGAAAGGTCGT-3' | 20 bp<br>20 bp | 108 bp | 27.71 | 27.25 | 27.72 |
| ***Prkar2b*** | NM_001030<br>020 | F: 5'-CCCATGCGCTCCGATTCCGA-3'<br>R:5'-GCACATACCGAGGCACGCCT-3' | 20 bp<br>20 bp | 107 bp | 31.51 | 29.87 | 31.13 |
| ***Gapdh*** | NM_017008 | F: 5'-TGCCAAGTATGATGACATCAAGAAG-3'<br>R: 5'-AGCCCAGGATGCCCTTTAGT-3' | 25 bp<br>20 bp | 110 bp | 21.85 | 21.57 | 21.03 |

Primers were designed by using software Primer Express 3.0 (Applied Biosystems) and full genes sequences from NCBI Entrez Nucleotide database (www.ncbi.nlm.nih.gov/sites/entrez). F - forward; R - reverse.

**Supplementary Table S8.** Primers sequences used for the real-time PCR analysis of MAPK signaling elements.

| Gene | Accession code | Primers | Primer length | Product length | AV Ct | | |
|---|---|---|---|---|---|---|---|
| | | | | | ZT3 | ZT3 | ZT3 |
| ***Mapk1*** | NM_053842.1 | F: 5'-GTTCTGCACCGTGACCTCAAG-3'<br>R: 5'-GCAAGGCCAAAGTCACAGATC-3' | 21 bp<br>21 bp | 80 bp | 28.11 | 26.84 | 28.00 |
| ***Mapk3*** | NM_017347.2 | F: 5'-TCCCTCTCAAGCTGCCACAT-3'<br>R: 5'-ACATCCAATCACCCACACACA-3' | 20 bp<br>21 bp | 60 bp | 27.56 | 26.90 | 27.01 |
| ***Mapk6*** | NM_031622.2 | F: 5'-CATTTGAACTGGCATGTCGTTT-3'<br>R: 5'-CCTGCACTGCATTGTTTTGC-3' | 22 bp<br>20 bp | 62 bp | 27.89 | 26.78 | 26.52 |
| ***Mapk7*** | NM_001191547.1 | F: 5'-GCCCCTTCCACTAGCCTTTT-3'<br>R: 5'-GAACCAGGCAACCCACTAGGT-3' | 20 bp<br>21 bp | 62 bp | 28.98 | 28.09 | 27.91 |
| ***Mapk8*** | NM_053829.2 | F: 5'-TCAACGTCTGGTATGATCCTTCA-3'<br>R: 5'- CTGCTTGTCAGGGATCTTTGG-3' | 23 bp<br>21 bp | 62 bp | 29.11 | 28.00 | 28.69 |
| ***Mapk9*** | NM_017322.1<br>NM_001270544.1<br>NM_001270545.1 | F: 5'-GGAAGGCTGCCGATGAAA-3'<br>R: 5'-AGCCAGAGTCCTTCACAGACAAG-3' | 18 bp<br>23 bp | 57 bp | 28.65 | 27.51 | 27.30 |
| ***Mapk11*** | NM_001109532.2 | F: 5'-GGGCGCTGACCTGAATAACA-3'<br>R: 5'-GCAGCAGCTGGTAGACAAGGA-3' | 20 bp<br>21 bp | 80 bp | 30.43 | 30.52 | 30.52 |
| ***Mapk12*** | NM_021746.1 | F: 5′-GGATGTGTTCACTCCCGATGA-3′<br>R: 5′-CCAGGTCAGTGCCCATGAAT-3′ | 21 bp<br>20 bp | 80 bp | 28.99 | 27.46 | 28.60 |
| ***Mapk13*** | NM_019231.2 | F: 5′-CTGGTCTGTTGGCTGCATCA-3′<br>R: 5′-TCAGCTGGTCCAGGTAGTCCTT-3′ | 20 bp<br>22 bp | 80 bp | 28.28 | 27.51 | 29.22 |
| ***Mapk14*** | NM_031020.2 | F: 5′-GCTGTCGACCTGCTGGAAAA-3′<br>R: 5′- TAGGCATGCGCAAGAGCTT-3′ | 20 bp<br>19 bp | 80 bp | 27.19 | 26.24 | 26.88 |
| ***Gapdh*** | NM_017008 | F:<br>5'-TGCCAAGTATGATGACATCAAGAAG-3'<br>R: 5'-AGCCCAGGATGCCCTTTAGT-3' | 25 bp<br>20 bp | 110 bp | 21.93 | 21.61 | 21.03 |

Primers were designed by using software Primer Express 3.0 (Applied Biosystems) and full genes sequences from NCBI Entrez Nucleotide database (www.ncbi.nlm.nih.gov/sites/entrez). F - forward; R - reverse.

**Supplementary Table S9.** The characteristics of the antibodies.

| Target | Name of antibody | Antigen sequence | Manufacturer, catalog # | Mono- or polyclonal | Dil. used |
|---|---|---|---|---|---|
| **PGC-1** | PGC-1 (H-300): sc-13067 | Amino acids 1-300 mapping near the N-terminus of PGC-1 of human origin | Santa Cruz Biotechnology Inc. sc-13067 Mw (PGC-1) = 90 kDa | Rabbit polyclonal antibody | 1:200 |
| **NRF2** | NRF2 (C-20): sc-722 | Peptide mapping at the C-terminus of Nrf2 of human origin | Santa Cruz Biotechnology Inc. sc-722 Mw (NRF2) = 57/100 kDa | Rabbit polyclonal antibody | 1:100 |
| **PKAc** | PKA [C]:610980 | Human PKA[Cα] subunit aa. 18-347 | BD Transduction Laboratories 610980 Mw (PKAc) = 40 kDa | Mouse monoclonal antibody | 1:200 |
| **p38MAPK** | p38MAPK: 9212 | Detects total p38$\alpha$, -ß or -$\gamma$ MAPK protein | Cell Signaling Technology 9212 Mw (p38MAPK) = 43 kDa | Polyclonal Rabbit antibody | 1:1000 |
